# Multifaceted, Brief Intensive Home-Based Exposure Treatment in Patients with Obsessive-Compulsive Disorder Who are Nonresponsive to Regular Cognitive Behavior Therapy: An Uncontrolled Pilot Study

**DOI:** 10.1097/PRA.0000000000000796

**Published:** 2024-07-26

**Authors:** Karin C.P. Remmerswaal, Neeltje M. Batelaan, Patricia van Oppen, Willemijn D. Scholten, Anton J.L.M. van Balkom

**Affiliations:** Department of Psychiatry, Amsterdam UMC Location Vrije Universiteit Amsterdam, Amsterdam Public Health Research Institute, and GGZ inGeest, Amsterdam, The Netherlands

**Keywords:** obsessive-compulsive disorder, cognitive behavior therapy, intensive treatment, concentrated treatment

## Abstract

**Objective:**

To test a multifaceted treatment program for patients with obsessive-compulsive disorder (OCD) who did not respond to regular cognitive behavior therapy (CBT). The treatment addresses several factors that may play a role in maintaining OCD.

**Methods:**

We designed a treatment consisting of a 6-day intensive, individual exposure in vivo with response prevention (ERP) format, with 24 therapist-assisted treatment hours at the patient’s home and 12 self-controlled ERP hours, including behavioral activation and family interventions. Next, we investigated the effect (obsessive-compulsive symptoms, comorbidity, functioning, quality of life, OCD-related interaction patterns) and feasibility (dropout, treatment satisfaction, and organization) of this program using pre-post-tests, pre-follow-up tests, and qualitative data from patients, family members, and therapists.

**Results:**

In a sample of 22 participants, obsessive-compulsive symptoms (Y-BOCS pre: 28.7, post: 15.9; *Wilcoxon S-R tests P*<0.01) improved significantly, as did most other effect measures. Results were largely, but not completely, preserved at 3-month follow-up. There was only 1 dropout. Patients, family members, and therapists were satisfied with the treatment. Implementation of the treatment did not pose difficulties.

**Conclusions:**

In nonresponders with OCD, a multifaceted, brief, intensive home-based ERP program targeting factors maintaining OCD is promising and feasible. Extra care is needed to maintain improvement.

Obsessive-Compulsive Disorder (OCD) follows a chronic course in the majority of patients.^1,2^ Moreover, it is a disabling disorder accompanied by widespread limitations in work, household activities, social relations and well-being.^3^ OCD also affects families of patients, as most relatives become involved in performing compulsions or in dysfunctional interactions with the patient related to the OCD.^4^

Cognitive behavior therapy (CBT) is a first-line, evidence-based treatment for OCD, with exposure to response prevention (ERP) being the main element in CBT treatment protocols.^5–7^ Treatment is most often provided in an individual format, with weekly sessions lasting 45 to 90 minutes each. However, about 40% to 55% of patients with OCD do not improve sufficiently with CBT.^6,8,9^ Possible causes of this nonresponse to CBT are a disturbed process of fear extinction, a lack of meaningful activities due to OCD, decreasing motivation during ERP, and dysfunctional interaction patterns with relatives related to OCD.

According to learning theory, exposure to a feared, conditioned stimulus (CS) can lead to the extinction of fear by patients perceiving that the CS is not accompanied by an aversive, unconditioned stimulus (US).^10^ However, a systematic review indicates that patients with OCD may have impairments in the fear extinction process, resulting in higher fear responses during extinction learning compared with healthy controls.^11^ This persistent fear can reinforce avoidance behavior and compulsions, thus acting to maintain OCD. To overcome these impairments, nonfear associations with CS may need to be strengthened and made available in more contexts for patients with OCD.^12^ It is hypothesized that this may be achieved through longer exposure sessions and consecutive sessions over a shorter period, with fewer interruptions from confrontations with the CS in daily life. One possible approach is a brief, intensive outpatient format of ERP, which involves substantially longer and more concentrated sessions than in a regular outpatient format. Examples are the Bergen 4-day treatment, which includes 25 hours of ERP in only 4 days,^13,14^ and a 5-day intensive treatment for children.^15^ Although research into such brief, intensive ERP is scarce, the available studies suggest it is a feasible and effective treatment approach.^16,17^ However, little is known about the effectiveness of brief intensive ERP for individuals who have not responded to regular ERP. A recent randomized controlled trial in anxiety disorders, excluding OCD, demonstrated that brief, intensive exposure was as effective as regular CBT in reducing symptoms and disabilities.^18^ Moreover, the concentrated approach resulted in a lower dropout rate and a faster treatment response compared with weekly exposure.^18^ A meta-analysis by Remmerswaal et al^16^ of studies involving brief, intensive exposure-related therapies in patients with anxiety disorders and OCD showed that brief, intensive exposure-based treatments did not differ significantly from regular treatments in terms of posttreatment outcomes. Brief intensive exposure-based treatment also led to quicker effects due to the shorter lead time.^16^ However, interpretation of the findings was challenging due to the limited studies available and their designs and quality.^16^ In an open trial of an intensive, family-based CBT in youths who did not respond or only partially responded to at least 2 medication trials, Storch et al^19^ found that 80% showed improvement after intensive CBT with a mean reduction of obsessive-compulsive symptoms of 54%.

Nonresponse to CBT may also be due to causes other than a disturbed process of fear extinction, such as a lack of meaningful daytime activities due to OCD. Although increasing activities with behavioral activation has been found to be effective in major depressive disorder,^20^ it has scarcely been studied in the context of OCD. A study published in 2017 found that improvements in functioning were related to a reduction in OCD symptom severity in patients with OCD treated with ERP and psychotropic medication.^21^ A 2018 study found that, after a 4-week intensive ERP treatment, the level of general functioning in patients with OCD was also associated with obsessive-compulsive symptom severity.^22^

Another reason for treatment nonresponse may be decreased motivation due to fatigue from exposure treatment.^23,24^ In addition, dysfunctional interaction patterns with family members with regard to OCD can be a reason for nonresponse, as patterns of accommodation (adapting, participating) and antagonism (opposing, criticizing) are related to poorer treatment outcomes.^25^

## OBJECTIVES

This pilot study had 2 goals. Our first goal was to evaluate the effects and the feasibility of a multifaceted, brief, intensive ERP treatment that was specially developed for patients with OCD who had not achieved adequate improvement through the first step of CBT. The treatment, which addressed several possible factors that may contribute to maintaining OCD, consisted of lengthy sessions of ERP in quick succession (to enhance fear extinction), sustaining motivation, resumption of meaningful activities, and improvement of family interaction patterns. To reduce the burden on family members, the treatment also involved broadening the social network of the patient. The study evaluated several factors such as obsessive-compulsive and comorbid symptoms, functioning, quality of life, family interaction patterns, patient and family satisfaction, therapist satisfaction, feasibility of the organization of the treatment, and dropout rates. A secondary aim of this study was to get an impression of the impact of the treatment on the course of OCD through consecutive case series using trajectories of day-to-day improvement.

## METHODS

### Participants and Procedure

Adult participants (≥18 y of age) with a principal DSM-5 diagnosis of OCD who had experienced no significant improvement after at least 1 recent CBT treatment for OCD, including weekly ERP, plus at least 1 pharmacological treatment (except for 3 patients who refused medication), and who had a score on the Yale-Brown Obsessive-Compulsive Scale (Y-BOCS)^26,27^ of at least 16 (indicating at least moderately severe obsessive-compulsive symptoms), were recruited between 2017 and 2021 from a mental health outpatient clinic specializing in OCD. Participants were included if they had a family member who agreed to participate in the treatment. Concomitant use of psychotropic drugs was allowed. Patients were on stable doses of medication at pretreatment. Before enrolling in the intensive exposure program, participants’ motivation was evaluated to ensure their willingness to engage in the treatment. Motivation was assessed on the basis of completion of preparatory homework (see below) and preparedness to engage in anxiety-provoking exercises without major reservations. OCD was diagnosed using the Structured Clinical Interview for DSM-IV (SCID-I).^28^ This pilot study was approved by the Medical Ethics Committee of the VU-University Medical Centre. All participants and their family members gave written informed consent.

### Multifaceted, Brief Intensive Exposure Treatment at Home

**FIGURE 1:**
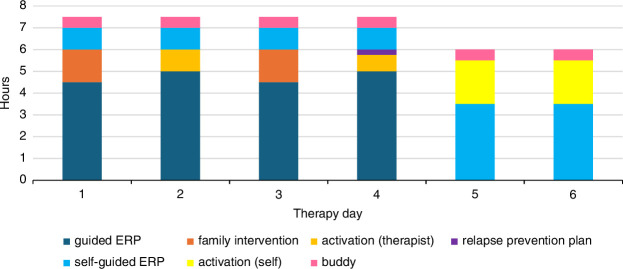
Schematic layout of treatment components.

The treatment consisted of 6 days with a total of 24 hours of therapist-guided therapy at the homes of the patients, consisting of 19 hours of ERP, 3 hours of family interventions, and 2 hours discussing activities. In addition, there were 12 hours of self-guided ERP, plus 3 follow-up sessions. For a schematic layout of the treatment, see Figure 1. The 6 treatment days were planned in 3 blocks of 2 consecutive days with breaks on the weekend and a day in between. Thus, the treatment had a 9-day lead time. The ERP-free days were scheduled so that the patient was able to recover from the intensive treatment days. The participants were instructed to engage in pleasurable activities while maintaining the results achieved.

The multifaceted, brief, intensive ERP had 4 main objectives. The first goal was to reduce the obsessive-compulsive symptoms of the patient through the use of ERP. The intensive ERP was delivered by 4 therapists who each provided treatment at the homes of the participants for a day (or at other places where the symptoms of OCD were most evident). The therapists shared their experiences with the therapist who was scheduled to work with the patient the following day. ERP included a total of 19 hours of therapist-assisted ERP and an additional 12 hours of self-controlled ERP. In addition, in the evenings after each treatment day, patients performed additional self-controlled ERP exercises besides the 12 hours mentioned above. The self-guided ERP exercises were designed to expand the number of situations in which the individuals developed a nonfear association, thereby preventing therapists from becoming “safety signals.” The content of the initial ERP exercises was based on the patient’s predominant compulsion and secondarily on other rituals practiced. ERP was delivered without hierarchy^29^ and conducted as behavior experiments. Thus, before ERP was started, the participant determined the feared consequence of the planned exercise. After ERP, the therapist and patient discussed whether the anticipated consequence had occurred and, if not, whether the obsession’s credibility had changed. Where the obsession could not be changed (eg, a “not just right” feeling after ERP that remained), the participant learned to resist responding to this obsession with compulsions. The second goal of the treatment was to normalize interactions related to OCD with the relatives closest to the patient, typically the partner or parents. There were 2 sessions of 90 minutes each with the patient and 1 or more family members to reduce accommodation and antagonism toward the patient’s obsessive-compulsive symptoms.^30^ In addition, patients purposefully contacted a buddy from their social network on a daily basis to discuss treatment and get support. This strategy can help increase understanding of OCD within the patient’s social circle, potentially reducing the burden and antagonizing behavior of family members. The buddy was instructed on how to support the patient, including psychoeducation on accommodation and antagonism, during a brief call with the therapist and the patient. The third goal of the treatment program was to help patients regain roles in daily life and society. Two sessions of 60 minutes each were spent discussing and planning (daily) activities and pleasurable activities, including those on the ERP-free days. The last goal was to prevent relapse. Patients created a personal relapse prevention plan, which was then reviewed and practiced in 3 follow-up sessions and through email correspondence.

After completing the 6 intensive ERP days at home, patients attended the first follow-up session of 45 minutes with the senior therapist who set up treatment. In this session, the therapist discussed the self-controlled ERP exercises with the patients and motivated them to continue practicing ERP exercises in the subsequent weeks. Their personal relapse prevention plan was also discussed. Furthermore, the patients were informed that they could contact the therapist in the case of relapse or crisis. After this, patients sent an email every 3 weeks with an update on their exposure exercises and symptoms. Two more follow-up sessions were scheduled with the patient and their family member at 1 and 3 months after the last session.

Before the start of the intensive treatment, several preparatory activities were conducted. Participants and a family member were invited for two 1-hour sessions with a senior therapist to determine the content of the intensive treatment. The participants received a patient manual which included instructions for making a case conceptualization and a list of exposure exercises. Patients also made an inventory of daily activities to resume after treatment. To remain motivated, the participants created a collage of their life without OCD and placed it in a central location in their home. In addition, participants asked someone from their social network to serve as a buddy to provide support and encouragement during the treatment period.

To ensure treatment integrity, a detailed day-to-day protocol was developed, and therapists underwent 2-hour training sessions. During the training, the treatment protocol was thoroughly discussed and therapists were trained in family interventions. This allowed for consistency in the application of the treatment across different therapists and ensured that patients received the same level of care and attention throughout their treatment.

### Assessments

Complete assessments were conducted pretreatment, posttreatment, and at 3-month follow-up; these included assessor-ratings by treatment-independent research nurses and self-ratings by patients and involved family members. Functioning and participation in society were assessed only at pretreatment and follow-up. In addition, daily self-report measures on the severity of OCD were completed by the patient during the 9-day lead time of the treatment and on the day before and the day after treatment. Finally, a questionnaire was completed by therapists at posttreatment. A detailed description of the instruments used is provided in online supplemental content, Supplemental Digital Content 1 (http://links.lww.com/JPP/A72).

#### Demographic and Clinical Characteristics

Data on the following demographic characteristics were gathered: age, gender, partner (yes, no), duration of relationship, children (yes, no), educational level, employment (yes, no), duration of OCD, presence of comorbid mental disorders, type and dosage of psychotropic medication, and number of previous treatments.

The severity of OCD was assessed using the original interview version of the Yale-Brown Obsessive-Compulsive Scale (Y-BOCS).^26,27^ A self-report version of the Y-BOCS (Y-BOCS-SR) was constructed for daily measurements that ask about the severity of obsessive-compulsive symptoms in the previous day. The number and content of the items of the original Y-BOCS were preserved as much as possible; only the requested time period was adjusted. The Y-BOCS-SR was used solely to study how the severity of symptoms changed day to day throughout treatment, while the original Y-BOCS was used to assess treatment outcomes. Also, the Padua Inventory-Revised (PI-R)^31^ self-report severity scale was filled out by the patient. Insight into OCD was measured by the Overvalued Ideas Scale (OVIS), a self-report questionnaire.^32^

Comorbid anxiety and depressive symptoms were measured using the Beck Anxiety Inventory (BAI)^33^ and the Beck Depression Inventory (BDI).^34^

Several measures were used to evaluate patients’ functioning and quality of life. The World Health Organization Disability Assessment Schedule-II (WHODAS)^35^ was employed to evaluate daily functioning, while the EuroQol 5-dimensional questionnaire (EQ-5D)^36^ was used to assess quality of life. In addition, objective and subjective participation in society was measured using the Utrecht Scale for Evaluation of Rehabilitation–Participation (USER-P).^37^

#### Relationship Between Person With OCD and Partner/Family: Measurements in Patients

To assess the perceived level of criticism by the family member, the Perceived Criticism Measure in the patient (PCM) was used.^38^ The Level of Expressed Emotion (LEE) was used to investigate the perceived level of expressed emotion from family members.^39^ Relationship satisfaction was measured using the Relationship Assessment Scale (RAS) only in those patients with a partner relationship.^40^ To assess the overall atmosphere at home, patients were asked to rate it on a visual analog scale ranging from 0 to 100, with higher scores meaning a more positive atmosphere.

#### Measurements in Partner/Family

The degree of accommodation to OCD by the partner/family members was measured using the Family Accommodation Scale–Interview version (FAS).^41^ Perceived level of criticism by the patient (PCM), relationship satisfaction (RAS), and atmosphere at home were also assessed. In addition, feelings of rejection toward the patient were measured using the Patient Rejection Scale (PRS).^42^ Daily functioning in the family member was measured using the WHODAS and quality of life with the EQ-5D.

#### Feasibility and Treatment Satisfaction

Dropout was defined as terminating treatment at any moment after completing the preparatory sessions. The level of satisfaction with the treatment among patients and their family members was assessed using a visual analog scale with values from 0 to 100, with higher values indicating more satisfaction. In addition, an interview consisting of 9 open-ended questions was conducted, which included questions such as “How did you experience the brief intensive ERP? What did you like/dislike about the program? How did you feel about dealing with multiple therapists?” The therapists’ satisfaction with the treatment was assessed using a questionnaire consisting of 7 open-ended questions, such as “How did you like working with this treatment? What did you think went well/not well?” The feasibility of the organization of the treatment was assessed by recording problems that arose in planning and organizing treatment (eg, problems in arranging therapists, unused scheduled treatment days).

### Statistical Analysis

Results were analyzed using the Statistical Package for Social Sciences, version 28.^43^ Missing values at posttreatment (*n*=2), at follow-up (*n*=5), and at the daily Y-BOCS-SR measurements (between *n*=4 and *n*=9) were imputed with multiple imputation, 50 times. Pooled results were reported. The baseline characteristics of the sample were described through means, SDs, numbers, and percentages. Results from pretreatment, posttreatment, and follow-up were analyzed using related-samples Wilcoxon signed-rank tests. If a value of *P*<0.05 was found in a majority of the imputed data sets, we considered the effect to be significant. Effect sizes ‘*r*’ were calculated by dividing the standardized test statistic by the square root of the number of observations, with 0.3 considered a medium effect size and 0.5 considered a large effect size.^44^ Jacobson and Truax’s method^45^ was used to determine reliable change on the Y-BOCS (≥4.5 decrease in score) and clinically significant recovery (CSR; reliable change and post-test Y-BOCS ≤18.9) at posttreatment and follow-up, using data from Frost et al^46^ and Kim et al.^47^ Furthermore, to examine differences in Y-BOCS outcomes in patients with and without posttreatment CSR, post hoc analyses were performed. Daily measurements of OCD severity were analyzed using linear mixed models (LMM) to examine whether improvement in OCD severity coincided with treatment days by comparing relative improvements from the previous day. This analysis was performed on the original, not the imputed, dataset, as LMM accounts for missing values.

## RESULTS

### Participants

Table 1 shows the baseline characteristics of our sample. A total of 22 participants (15 female; 68%) were included in this pilot study, with a mean age of 37.2 years (SD=13.6 y) and a mean duration of OCD of 17.5 years (SD=11.9 y). Patients were well educated: 10 (45%) had finished general secondary education or secondary vocational education and 12 (55%) had finished higher professional education or university.

**TABLE 1:** Description of Sample at Baseline (*N*=22)

	Mean±SD or n (%)
Sociodemographic characteristics	
Age (y)	37.2±13.6
Gender, female, *n* (%)	15 (68)
Partner, yes, *n* (%)	15 (68)
Duration partner relationship (y)	12.7±13.7
Child(ren), yes, *n* (%)	7 (32)
Education, *n* (%)	
General secondary education/secondary vocational education	10 (45)
Higher professional education/university	12 (55)
Working (paid job), yes	12 (55)
Clinical characteristics	
Duration of OCD (y)	17.5±11.9
Comorbid disorders, yes, *n* (%)	14 (64)
Psychotropic medication, yes, *n* (%)	17 (77)
Number of previous treatments	
Psychotherapy	3.2±2.4
Psychotropic medication	2.1±2.3

One participant (5%) reduced the dose of antidepressants during the intensive treatment phase of the study due to adverse effects. During the follow-up period, 16 patients (73%) continued to follow the protocol of the brief intensive home-based ERP and had no medication changes, while 6 patients (27%) required additional treatment or a medication change: 3 patients received psychotherapy and psychotropic medication because of depressive symptoms, 1 participant received group sessions for OCD, 1 patient increased the dose of medication, and 1 patient stopped medication.

The baseline assessments (Table 2) suggest that the sample under study had severe obsessive-compulsive symptoms (Y-BOCS mean score of 28.7, SD=5.1) with fair insight (OVIS mean score of 4.9, SD=1.2). All measured OCD subtypes were present. In addition, participants scored moderately high on comorbid anxiety and depressive symptoms. The participants in our sample had a relatively poor quality of life, and they had a higher level of disabilities than 90% of the general population. Participants experienced many restrictions in participation in society and were not satisfied with their level of participation, although the frequency of their activities was found to be comparable to that of a reference group of rehabilitation outpatients who were physically or cognitively independent. Although the patients in the current study reported a moderate general level of expressed emotion in the relationship with the family member closest to them, they perceived the family members as critical. The atmosphere at home was adequate. Those with a partner (*n*=15) were satisfied with their relationship.

**TABLE 2:** Treatment Outcome of Intensive ERP at Home in a Pilot Sample of 22 Participants with OCD Nonresponsive to Previous CBT

	Mean±SD			P-range Wilcoxon S-R Tests No. Imputation*s P<0.05* r	
	Pretreatment	Posttreatment	Follow-up^*^	Pre–post	Pre–follow-up
Y-BOCS^†^ (range 0-40)	28.73±5.10	15.87±7.33	17.50±7.35	<0.001	<0.001
				50^‡^	50^‡^
				−0.61	−0.60
PI-R^§^ (range 0-164)	71.86±28.66	41.87±23.92	51.85±31.40	<0.001	<0.001
				50^‡^	50^‡^
				−0.60	−0.51
Impulses∥ (range 0-28)	4.50±3.54	2.59±2.79	3.27±2.87	<0.001-0.044	0.016-0.137
				50^‡^	15
				−0.42	−0.28
Washing∥ (range 0-40)	17.64±14.30	10.10±10.19	11.95±11.79	<0.001	0.005-0.006
				50^‡^	50^‡^
				−0.55	−0.43
Checking∥ (range 0-28)	13.82±7.92	6.93±5.74	9.49±6.93	<0.001	0.002-0.021
				50^‡^	50^‡^
				−0.56	−0.42
Rumination∥ (range 0-44)	25.18±8.52	17.35±7.88	20.32±9.64	<0.001-0.002	0.004-0.028
				50^‡^	50^‡^
				−0.50	−0.39
Precision∥ (range 0-24)	10.73±6.44	4.95±5.22	6.83±5.84	<0.001	0.001-0.003
				50^‡^	50^‡^
				−0.58	−0.48
OVIS^¶^ (range 0-10)	4.92±1.15	3.66±1.60	4.10±1.58	0.001-0.012	0.009-0.127
				50^‡^	39^‡^
				−0.44	−0.32
BAI^#^ (range 0-63)	23.86±10.79	15.50±10.55	15.61±13.14	<0.001-0.001	<0.001-0.001
				50^‡^	50^‡^
				−0.52	−0.56
BDI^**^ (range 0-63)	20.68±8.86	15.26±9.42	15.91±11.75	0.001-0.033	<0.001-0.021
				50^‡^	50^‡^
				−0.56	−0.45
WHODAS^††^ (range 0-100)	41.47±12.10	NA	34.67±15.29	NA	0.011-0.046
					50^‡^
					−0.35
EQ-5D^‡‡^ (range 0-1)	0.47±0.29	0.74±0.28	0.73±0.28	<0.000-0.028	<0.001-0.065
				50^‡^	48^‡^
				0.47	0.45
User-Pfrequencies^§§^ (range 0-100)	31.62±8.86	NA	33.27±9.37	NA	0.148– 0.548
					0
					0.18
User-P restrictions∥∥ (range 0-100)	66.71±17.59	NA	75.19±17.89	NA	0.020–0.060
					44^‡^
					0.31
User-P satisfaction¶¶ (range 0-100)	52.27±14.58	NA	65.56±15.33	NA	0.002-0.009
					50^‡^
					0.43
PCM## (range 1-10)	5.41±2.79	3.89±2.14	4.22±2.63	0.010-0.030	0.003-0.040
				50^‡^	50^‡^
				−0.36	−0.37
LEE^***^ (range 0-114)	66.82±19.18	61.26±13.35	64.04±16.60	0.273-0.375	0.820-1.000
				0	0
				−0.16	0.00
RAS^†††^ (range 1-5)	4.48±0.52	4.43±0.53	4.42±0.61	0.088-1.000	0.251-0.968
				0	0
				−0.09	−0.09
Atmosphere at home^‡‡‡^ (range 0-100)	65.27±26.74	78.04±10.10	73.78±13.12	0.026-0.044	0.211-0.271
				50^‡^	0
				0.32	0.17
Satisfaction w/treatment^§§§^ (range 0-100)	NA	82.60±14.67	NA	NA	NA

^*^ Follow-up after 3 months.

^†^ Y-BOCS: higher scores indicate greater severity of symptoms.

^‡^ *P*<0.05 in >25 imputed data sets.

^§^ PI-R: clinical cut-off ≥53.

^∥^ Subscales of the PI-R.

^¶^ OVIS: ≥6 indicates poor insight.

^#^ BAI: higher scores indicate more severe anxiety.

^**^ BDI: higher scores indicate more severe depression.

^††^ WHODAS: higher scores indicate more disabilities.

^‡‡^ EQ-5D: assesses health status/quality of life; 1 indicates best possible health status.

^§§^ User-P—Frequencies: higher scores indicate greater levels of participation in life activities.

^∥∥^ User-P—Restrictions: higher scores indicate lower levels of limitations in daily life.

^¶¶^ User-P—Satisfaction: higher scores indicate greater satisfaction with activities and life.

^##^ PCM: higher scores indicate greater levels of criticism.

^***^ LEE: higher scores indicate higher levels of expressed emotion.

^†††^ RAS: higher scores indicate more satisfaction in the relationship.

^‡‡‡^ Higher scores indicate a more positive atmosphere.

^§§§^ Higher scores indicate greater satisfaction.

BAI indicates Beck Anxiety Inventory; BDI, Beck Depression Inventory; EQ-5D, EuroQol 5-Dimensional Questionnaire; LEE, Level of Expressed Emotion; NA, not applicable; OVIS, Overvalued Ideas Scale; PCM, Perceived Criticism Measure; PI-R, Padua Inventory-Revised; RAS, Relationship Assessment Scale, *n*=15; User-P, Utrecht Scale for Evaluation of Rehabilitation–Participation; WHODAS, World Health Organization Disability Assessment Schedule 2.0; Y-BOCS, Yale-Brown Obsessive-Compulsive Scale.

Family members (Table 3) accommodated the obsessive-compulsive symptoms to a moderate degree. Significant antagonism toward and rejection of the patient were present, as assessed by the Perceived Criticism Measure and the Patient Rejection Scale. Despite this, partners were satisfied with the relationship with the patient and experienced the atmosphere at home as rather good. The functioning and quality of life of family members were at the level of the general population.

**TABLE 3:** Treatment Outcome of Intensive ERP at Home in a Pilot Sample of 22 Family Members of Participants with OCD Nonresponsive to Previous CBT

	*Mean*±*SD*			*P-*range Wilcoxon *S-R* tests No. Imputations *P<0.05* *r*	
	Pretreatment	Posttreatment	Follow-up^*^	Pre–post	Pre–follow-up
FAS^†^ (range 0-48)	15.17±7.11	3.44±5.30	2.60±3.13	<0.001	<0.001
				50^‡^	50^‡^
				−0.54	−0.62
PCM^§^ (range 1-10)	4.94±2.63	4.46±2.31	4.28±1.73	0.107-0.931	0.019-0.757
				0	8
				−0.13	−0.21
PRS∥ (range 11-33)	23.36±2.86	22.75±1.34	23.41±1.80	0.034-0.983	0.159-1.000
				1	0
				−0.14	0.07
RAS^¶^ (range 1-5)	4.16±0.61	4.39±0.40	4.21±0.46	0.006-0.582	0.222-1.000
				24	0
				0.35	0.01
Atmosphere at home^#^ (range 0-100)	73.71±14.34	82.23±8.34	81.49±8.63	0.002-0.014	<0.001-0.009
				50^‡^	50^‡^
				0.44	0.46
WHODAS^**^ (range 0-100)	9.58±9.88	NA	4.57±3.83	NA	0.01-0.051
					49^‡^
					−0.40
EQ-5D^††^ (range 0-1)	0.89±0.23	0.97±0.07	0.95±0.10	0.011–1.000	0.015-0.953
				17	3
				0.26	0.16
Satisfaction with treatment^‡‡^ (range 0-100)	NA	82.56±8.52	NA	NA	NA

^*^ Follow-up after 3 months.

^†^ FAS: higher scores indicate higher levels of accommodation and greater consequences of not accommodating.

^‡^ *P*<0.05 in >25 imputed data sets.

^§^ PCM: higher scores indicate greater levels of criticism.

^∥^ PRS: higher scores indicate a greater degree of rejection.

^¶^ RAS: higher scores indicate more satisfaction in the relationship.

^#^ Higher scores indicate a more positive atmosphere.

^**^ WHODAS: higher scores indicate more disabilities.

^††^ EQ-5D: assesses health status/quality of life; 1 indicates best possible health status.

^‡‡^ Higher scores indicate greater satisfaction.

EQ-5D indicates EuroQol 5-dimensional questionnaire; FAS, Family Accommodation Scale; NA, not applicable; PCM, Perceived Criticism Measure; PRS, Patient Rejection Scale; RAS, Relationship Assessment Scale (n = 15); WHODAS, World Health Organization Disability Assessment Schedule 2.0

### Outcomes

#### Clinical and Relationship Measures

Table 2 presents the pretreatment, posttreatment, and follow-up test scores of the patients. The severity of obsessive-compulsive symptoms decreased significantly in this group of patients who had previously not responded to treatment. As measured using the classical Y-BOCS, 82% of the patients achieved a reliable improvement, while 67% met the criteria for recovery at posttreatment, according to the method of Jacobson and Truax.^45^ However, these outcomes were not fully preserved at 3-month follow-up, when 76% of patients remained reliably improved, and 48% of patients remained recovered. Insight into OCD improved significantly, just as the severity of comorbid depressive and anxiety symptoms decreased and functioning and quality of life improved. The level of activity, relationship satisfaction, and the level of expressed emotion did not change significantly according to the patients. The atmosphere at home improved significantly after treatment, although this effect was not preserved at follow-up. Antagonism, which was assessed by a different measure, decreased significantly, although it was still severe, according to the patient questionnaire.

The results from the family members are presented in Table 3. Accommodation of family members to the obsessive-compulsive symptoms of the patients decreased significantly and was more or less absent at posttreatment and follow-up. Antagonism and rejection of the patient, according to the family members, however, were not significantly decreased and remained severe. The satisfaction of partners with the relationship with the patient did not change significantly and remained good. The atmosphere at home improved significantly to good. The functioning of the family members improved significantly as well, but the quality of life did not. However, the relatives’ quality of life was already at the community level at pretreatment.

**FIGURE 2:**
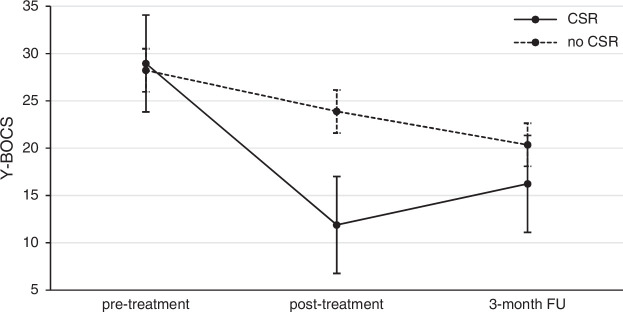
Treatment outcome associated with severity of OCD in patients with (n=14.8) and without (n=7.2) a clinically significant recovery (CSR).

Figure 2 presents the results of the Y-BOCS for patients with and without a clinically significant recovery (CSR) at posttreatment. In 39 of the 50 imputed data sets, posttreatment scores on the Y-BOCS were estimated so that 15 participants had a CSR; in 10 of the imputed data sets, 14 participants had a CSR; and in 1 dataset, 13 participants had a CSR, resulting in a pooled number of participants with a CSR of 14.8. Recovered patients had a significant increase in obsessive-compulsive symptoms from posttreatment to follow-up (posttreatment: mean=11.88, SD=3.66; follow-up: mean=16.22, SD=5.07; Wilcoxon S-R test *P-*range [0.002, 0.023]; *P*<0.05 in all imputed data sets). In contrast, symptoms decreased further at follow-up in patients without a CSR, but this effect was not significant (posttreatment: mean=23.88, SD=6.25; follow-up: mean=20.36, SD=10.64; Wilcoxon S-R test *P-*range [0.237, 0.735]; *P*≥0.05 in all imputed data sets). The results shown in Figure 2 suggest that patients with and without CSR did not differ in whether they responded to treatment at all, but rather that their response patterns differed.

#### Daily Measurements of the Severity of OCD

**FIGURE 3:**
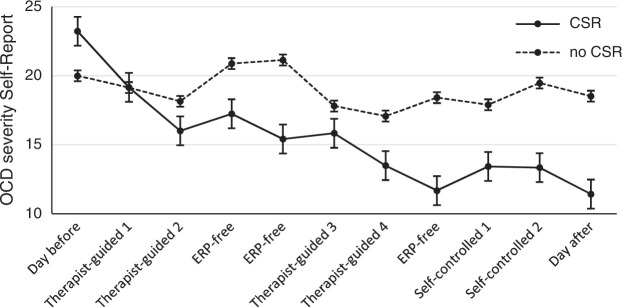
Course of the severity of OCD during treatment according to patients with (n=14.8) and without (n=7.2) a clinically significant recovery (CSR).

During treatment, the severity of OCD was measured daily with a self-report version of the Y-BOCS (see Methods section). The severity of OCD decreased significantly during the 9-day treatment period (*Beta*=0.49; *P*=0.01). The course of OCD severity revealed an early response: after 2 treatment days when 65% of the total effect was achieved (5.45 of 8.41 points total improvement on the Y-BOCS-SR). Moreover, the degree of improvement after 2 treatment days was significantly correlated with a better posttreatment outcome as measured with the assessor-rated Y-BOCS (Spearman’s *ρ*=0.54; *P*=0.02). However, the outcome at follow-up was not correlated with the response after 2 treatment days (Spearman’s ρ=0.19; P=0.44). Post hoc analyses revealed that, compared with ERP-free and nontreatment days, the decrease in obsessive-compulsive symptoms was significantly greater after therapist-guided treatment days (*Beta*=−3.02, *P*=0.01), but not after self-guided ERP days (*Beta*=0.16, *P*=0.98). The results of the daily measurements are shown in Figure 3.

#### Feasibility and Satisfaction

Regarding dropouts, 1 participant whose baseline assessments were included in the analyses and whose missing measurements were imputed was unable to start treatment because of COVID-19 symptoms in combination with a difficult situation at home. She, therefore, started another treatment. In addition, 1 patient refused to participate in the posttreatment and follow-up measurements for unknown reasons, although this patient did complete the treatment program.

The patients’ level of satisfaction with the treatment was high (mean=82.60; SD=14.67). Qualitative data showed that the patients appreciated the individual format, the therapists, whom they experienced as being professional and kind, the fact that the treatment took place at their homes and that they could not easily avoid participating. The qualitative data also showed that the patients were happy with the results. The patients also appreciated that the therapists pushed only for exposure that was within the patients’ limits. In addition, they were satisfied with the multiple therapists; they reported that the therapists each had their own style and that they learned different aspects of ERP from each therapist. However, patients thought that the treatment was demanding and exhausting, and they expressed a desire for more posttreatment support.

Family members of the patients also expressed high levels of satisfaction with the treatment (mean=82.56; SD=8.52) for the same reasons mentioned by the patients. In addition, they appreciated learning strategies to help them deal in the best way with the OCD of their loved ones. However, it was challenging for them to see the patients being anxious and tired due to the treatment, and this made them feel powerless and drained. Family members also expressed a desire for more aftercare.

Information from the therapists concerning their satisfaction with the treatment was available for 68 of the total 84 treatment days. In summary, therapists reported feeling highly motivated to work with the brief intensive ERP due to the valuable insight they gained into the symptoms and the suffering of the patient, as well as the significant progress achieved. According to the therapists, they valued the chance to work together with other therapists, which made it simpler to adhere to the treatment plan. However, therapists found providing the intensive treatment to be quite exhausting. In addition, therapists recommended reducing the duration of the therapy sessions and increasing aftercare. A discussion of outcomes related to the feasibility of the treatment’s organization is provided in the supplemental digital content 1 (http://links.lww.com/JPP/A72).

## DISCUSSION

Our multifaceted, brief, intensive exposure treatment at home had an impact on several possible factors that maintain OCD. The treatment involved: (i) a series of lengthy ERP sessions in quick succession, plus a motivation preservation intervention to overcome possible problems regarding fear extinction and decreasing motivation to achieve a reduction in OCD symptoms (goal 1); (ii) sessions with family members and installing a buddy to normalize OCD-related interaction patterns with relatives (goal 2); (iii) re-establishing meaningful and pleasurable daytime activities to recover roles in daily life and in society (goal 3); and (iv) relapse prevention (goal 4). This treatment appeared effective and feasible in our sample of patients with severe and chronic OCD with many comorbidities and an extensive treatment history. In addition, the pre-set goals of this treatment were largely met. The severity of OCD decreased significantly, as did accommodation by relatives. Antagonism significantly decreased as well, even though the scores still indicated severe antagonism. Patients also reported a significant improvement in their functioning, quality of life, and satisfaction with their participation, along with a decrease in perceived restrictions on social participation. However, although improving level of activity was a preset treatment goal, it did not significantly increase from pretreatment levels. However, it turned out that level of activity was already adequate at baseline. Results were not completely maintained at follow-up, although 76% of the patients continued to be reliably improved and 48% remained recovered. Only 1 patient did not participate in the treatment due to personal circumstances, and patients, their family members, and therapists all expressed much satisfaction with the treatment, indicating good feasibility for this treatment format. By planning it ad hoc with 4 different therapists, treatment was also easily implemented in our outpatient clinic. The course of OCD during treatment and follow-up suggested that patients had differential response patterns.

The multifaceted approach is a promising treatment for patients with OCD who have not responded to regular CBT. This is an important finding, as few treatments are available for these patients. Due to a short lead time, results are obtained in a short time, thereby reducing suffering more quickly than in weekly treatment formats, allowing patients to resume work, education, and care duties sooner. Moreover, the costs of this treatment are the same as those of a weekly format because the number of sessions is the same, only the planning is different. There are several possible explanations for why the multifaceted brief, intensive exposure treatment was effective in these patients while previously delivered regular CBT was not. First, the lengthy ERP sessions in quick succession may have facilitated fear extinction. Second, CBT commonly consists of self-guided exposure, whereas in the brief intensive treatment, the exposure was therapist-assisted. Congruent with this explanation, the self-controlled exposure days in our study did not yield any symptom reduction compared with the therapist-assisted sessions. This observation is supported by a 1996 meta-analysis^48^ and an RCT published in 2007,^49^ which found that therapist-guided ERP was more effective than self-controlled ERP. However, an RCT published in 2010 did not find significant differences between the two treatment formats.^50^ Third, ERP was offered in a context in which the patients typically perform rituals, which may have increased the effectiveness of the treatment. Thus, therapists went to the homes of patients, outside, or to shops. Fourth, therapy was performed by 4 therapists, thereby introducing a variability of styles, approaches, and contexts. This might enhance the chance that an approach is offered that appeals to the patient. Another possibility is that each therapist starts with fresh energy. Fifth, therapists may have adhered to the treatment protocol better than they might otherwise have done because of the availability of a detailed manual and the collaboration with colleague therapists. Sixth, family members stopped accommodating the patient’s symptoms completely. Last, we selected highly motivated patients who, beforehand, were willing to perform exposure exercises that triggered a lot of fear. If less motivated patients had also been included, the results might have been less favorable, and the dropout rate might have been higher.

It is noteworthy that in patients who had recovered, obsessive-compulsive symptoms significantly increased in the follow-up period. The results at follow-up may even suggest an overly favorable view of the brief intensive exposure treatment due to the extra care that about one-quarter of the sample (5/22) received while participating in this study (see Results–Participants). The increase in obsessive-compulsive symptoms during follow-up could be related to a lack of increase in the patient’s participation in society or due to a lack of improvement in the amount of criticism expressed in the patient’s home or in the family members’ acceptance of the patient. These factors may increase the risk of relapse.^22,25^ Our results may imply that aftercare needs to be improved in those who recovered to prevent the worsening of symptoms, as mentioned by the therapists, patients, and family members. Improving emotional interaction at home and rehabilitation, where patients are stimulated to take up hobbies and responsibilities at home and in society, may be part of aftercare.

The degree of symptom improvement after 2 treatment days was significantly correlated with a better posttreatment outcome, suggesting that it is important to achieve results at the beginning of treatment. In addition, this result could mean that the response to treatment was rapid. However, the outcome at 3-month follow-up was no longer correlated with an early improvement. Thus, a rapid response should not be used as an early predictor of treatment response. A rapid response to treatment as a predictor of treatment success has previously been found in patients with OCD in residential treatment.^51^ However, this study did not include follow-up measurements.^51^ A rapid response has also been studied in patients with other mental disorders, such as anxiety disorders, depression, somatoform disorders, body dysmorphic disorder, and eating disorders, and it was found to be related to treatment outcomes in most studies.^52–56^ In OCD, sudden gains have been studied as well—defined as a large, rapid, and stable decrease in symptoms during treatment, not necessarily at the beginning of treatment—with mixed results, leaving unclear the importance of sudden gains in the treatment of OCD.^57–59^ The current study suggests that differential patterns of response to treatment are present, namely a rapid response pattern with better posttreatment outcomes but prone to relapse and a slow response pattern, with smaller improvements at posttreatment, but with continuing slow progression after treatment. These response patterns were not associated with the OCD subtype in this study (data not shown), as was suggested in a previous study,^57^ and they may instead reflect the presence of differential reinforcers of OCD, which react at different rates. For example, fear in those with a rapid response pattern, and habit or dysfunctional cognitions in slow response patterns.

A strength of this study was that it was the first to investigate brief intensive ERP in patients who were nonresponsive to regular CBT. The results offer hope for this group of patients. Another strength is the use of a naturalistic sample of patients with severe and chronic OCD who did not respond to regular ERP. Hence, our findings can be generalized to this population. However, a limitation of this study is that we did not have a control group. Therefore, it is not known whether the outcomes are related to the treatment, and results should be cautiously interpreted. Nonetheless, the results are unlikely to be due to other factors, as the patients had chronic OCD and had previously undergone several treatments with insufficient results. Another limitation is the inability to determine which specific elements of treatment led to the observed reduction in symptoms. The reduction of symptoms, however, coincided in time with therapist-guided treatment days.

The effectiveness of the brief, intensive ERP needs to be substantiated with an RCT. In addition, future research might focus on determining for which patients this treatment is most suitable. Because of its clinical relevance, it is also recommended that differential response patterns to CBT be investigated. Finally, research should examine how results that have been achieved can be better maintained.

## Supplementary Material

SUPPLEMENTARY MATERIAL
