# Supplementary material for: Multifaceted, Brief Intensive Home-Based Exposure Treatment in Patients with Obsessive-Compulsive Disorder Who are Nonresponsive to Regular Cognitive Behavior Therapy: An Uncontrolled Pilot Study
Source: J Psychiatr Pract. 2024 Jul 26;30(4):297–307. doi: 10.1097/PRA.0000000000000796 (PMC11280449; doi:10.1097/PRA.0000000000000796)
Supplement: SUPPLEMENTARY MATERIAL [file pra-30-297-s001.docx]

**Supplement Digital Content**

**Multifaceted, Brief Intensive Exposure Treatment at Home in Patients With Obsessive-Compulsive Disorder Who Are Nonresponsive to Regular Cognitive Behaviour Therapy: A Pilot Study.**

Karin C.P. Remmerswaal, PhD

Neeltje M. Batelaan, MD, PhD

Patricia van Oppen, PhD

Willemijn D. Scholten, PhD

Anton J.L.M. van Balkom, MD, PhD

Amsterdam UMC location Vrije Universiteit Amsterdam, Department of Psychiatry, Amsterdam Public Health research institute, and GGZ inGeest, Amsterdam, The Netherlands

**DESCRIPTION OF MEASUREMENT INSTRUMENTS**

The Yale-Brown Obsessive-Compulsive Scale (Y-BOCS) is a clinical interview consisting of 10 items that are scored on a 5-point Likert scale assessing the severity of obsessive-compulsive symptoms in the previous week. Total scores range from 0 to 40 (0-7: subclinical; 8-15: mild; 16-23 moderate; 24-31 severe; 32-40: extreme). The validity and reliability of the Y-BOCS are good.^1,2^

The Padua Inventory–Revised (PI-R)^3^ consists of 41 items that are scored on a 5-point Likert scale. The total score ranges from 0 to 164. The clinical cut-off score is 53 and higher. The PI-R has 5 subscales: impulses (fear of losing control over oneself; 7 items, subscale range 0-28); washing (contamination obsessions and washing compulsions; 10 items, range 0-40); checking (checking compulsions; 7 items, range 0-28); rumination (obsessive worrying and doubting; 11 items, range 0-44); and precision (grooming rituals and compulsions with numbers; 6 items, range 0-24). The PI-R has good to excellent psychometric properties.

The Overvalued Ideas Scale (OVIS)^4,5^ consists of 10 items, each with a range from 0 to 10 assessing the severity of overvalued ideation in the previous week. The total score is the mean of all item scores with higher scores representing poorer insight (< 6: good to moderate insight; ≥ 6: poor insight). The reliability and validity of the OVIS are adequate.

The Beck Anxiety Inventory (BAI)^6–8^ consists of 21 items assessing the severity of anxiety symptoms in the previous week on a 4-point Likert scale. Scores range from 0 to 63 (0-7: minimal; 8-15: mild; 16-25 moderate; 26-63 severe). The BAI has good psychometric properties.^6^

The Beck Depression Inventory (BDI)^9,10^ consists of 21 items assessing the severity of depressive symptoms in the previous week on a 4-point Likert scale. The total score ranges from 0-63 (0-13: minimal; 14-19: mild; 20-28: moderate; 29-63: severe). The psychometric properties of the BDI are good.^9^

The World Health Organization Disability Assessment Schedule-II (WHODAS) consists of 36 items on cognition, mobility, self-care, getting along, life activities, and participation. The total score is converted to a standardized score ranging from 0 to 100, with higher scores reflecting more disabilities in the previous 30 days. The psychometric properties of the WHODAS are good.^11^

The EuroQol five dimensional questionnaire (EQ-5D) contains 5 dimensions: mobility, self-care, daily activities, pain/discomfort, and depression/anxiety. Each dimension is rated at 3 levels in conformity with the health condition on the day of assessment: no problems, some problems, or major problems. The health states are converted into an index score—the EQ-5D—reflecting the generic overall QoL that can be used to compare QoL in various conditions. The EQ-5D has a value between 0 (worst possible health) and 1 (best possible health). The EQ-5D has been proven reliable and valid, and feasible to use.^12–15^

The Utrecht Scale for Evaluation of Rehabilitation–Participation (USER-P)^16,17^ consists of 3 scales: (i) frequency of participation, experienced; (ii) restrictions in participation, and (iii) satisfaction with participation. The frequency scale consists of 11 items, including 4 items assessing the number of hours per week spent on work, education, and household duties, and 7 items assessing the frequency of leisure and social activities in the previous 4 weeks on a 6-point Likert scale. The restrictions scale consists of 11 items assessing limitations in daily life due to the present disorder on a 4-point Likert scale. The satisfaction scale consists of 10 items assessing the satisfaction with daily activities and life on a 5-point Likert scale. Scale scores are converted to a standardized score ranging from 0 to 100, with higher scores indicating better participation (higher frequency, less restrictions, higher satisfaction). The USER-P is valid and reliable.^16,17^

The Perceived Criticism Measure (PCM) consists of 1 item: ‘How critical is your partner of you?’ and has a score between 1 and 10, with higher scores representing more criticism. The cut-off score for much criticism is 4 and above. The psychometric properties of the PCM are good.^18,19^

The Level of Expressed Emotion (LEE)^20,21^ consists of 38 items that are scored on a 4-point Likert scale. The total score ranges from 0 to 114 and expresses the emotional climate as perceived by the patient in the relationship with the closest family member. Higher levels of expressed emotion are related to poorer treatment outcome and relapse.^22^

The Relationship Assessment Scale (RAS)^23,24^ consists of 7 items that are scored on a 5-point Likert scale. The total score is the mean of all item scores, ranging from 1 to 5, with higher scores reflecting more satisfaction. Validity and reliability of the RAS are good.^23,24^

The Family Accommodation Scale (FAS) is a clinical interview assessing accommodation to the patient’s obsessive-compulsive symptoms in the previous week. It consists of 12 items that are scored on a 5-point Likert scale. Total scores range from 0 to 48 (0-12: mild; 13-24 moderate; 25-36 severe; 37-48 extreme). The reliability and validity of the FAS are good.^25^ The FAS has two subscales (accommodation, consequences of not accommodating) and a total score.

The Patient Rejection Scale (PRS) consists of 11 items that are scored on a 3-point Likert scale. The total score ranges from 11 to 33, with higher scores indicating a greater degree of rejection. Psychometric properties are satisfactory.^26,27^

**OUTCOMES**

Only 2 treatment days (of a total of 84 sessions) were cancelled by a therapist once the treatment had started, due to personal circumstances. None of the participants cancelled treatment days. Initially, therapists reserved time slots in their calendar to schedule a brief intensive ERP treatment day. However, these time slots were generally left unused. Moreover, in order not to waste therapist hours, patients were sometimes invited for the treatment before they had completed the preparatory homework, which seemed to diminish the patient's awareness of its importance. For these reasons, the planning was changed into an ad hoc planning whereby therapists only scheduled sessions once a patient was accepted for the treatment and had completed the preparatory work. It was possible to plan all treatments within 6 weeks after inclusion without difficulties and without any loss of productive hours of therapists. During the Covid-19 pandemic however, the brief intensive ERP could not be executed.

**REFERENCES**

1. Goodman W, Price LH, Rasmussen SA, et al. The Yale-Brown Obsessive Compulsive Scale. II. Validity. *Arch Gen Psychiatry*. 1989;46:1012–1016.

2. Goodman W, Price LH, Rasmussen SA, et al. The Yale-Brown Obsessive Compulsive Scale. I. Development, use, and reliability. *Arch Gen Psychiatry*. 1989;46:1006–1011.

3. Sanavio E. Obsessions and compulsions: the Padua inventory. *Behav Res Ther*. 1988;26:169–177.

4. Neziroglu F, McKay D, Yaryura-Tobias JA, et al. The overvalued ideas scale: development, reliability and validity in obsessive-compulsive disorder. *Behav Res Ther*. 1999;37:881–902.

5. Neziroglu F, Stevens KP, McKay D, et al. Predictive validity of the overvalued ideas scale: outcome in obsessive-compulsive and body dysmorphic disorders. *Behav Res Ther*. 2001;39:745–756.

6. Beck A, Epstein N, Brown G, et al. An inventory for measuring clinical anxiety: psychometric properties. *J Consult Clin Psychol*. 1988;56:893–897.

7. Gillis MM, Haaga DAF, Ford GT. Normative values for the Beck Anxiety Inventory, Fear Questionnaire, Penn State Worry Questionnaire, and Social Phobia and Anxiety Inventory. *Psychol Assess*. 1995;7:450–455.

8. Osman A, Barrios FX, Aukes D, et al. The Beck Anxiety Inventory: psychometric properties in a community population. *J Psychopathol Behav Assess.* 1993;15:287–297. doi:10.1007/BF00965034

9. Beck AT, Steer RA, Carbin MG. Psychometric properties of the Beck Depression Inventory: twenty-five years of evaluation. *Clin Psychol Rev*. 1988;8:77–100. doi:10.1016/0272-7358(88)90050-5

10. Roelofs J, Van Breukelen G, De Graaf LE, et al. Norms for the Beck Depression Inventory (BDI-II) in a large Dutch community sample. *J Psychopathol Behav Assess*  2012;35:93–98. doi:10.1007/S10862-012-9309-2

11. Üstün T, Kostanjsek N, Chatterji S, et al. *Measuring Health and Disability : Manual for WHO Disability Assessment Schedule (‎WHODAS 2.0)‎*. World Health Organization; 2010. Accessed November 10, 2022. https://www.who.int/publications/i/item/measuring-health-and-disability-manual-for-who-disability-assessment-schedule-(-whodas-2.0)

12. EuroQol Group. EuroQol—a new facility for the measurement of health related quality of life. *Health Policy (New York)*. 1990;(16):199–208.

13. Van Agt H, Essink-Bot ML, Krabbe P, et al. Test-retest reliability of health state valuations *collected* with the EuroQol questionnaire. In: Kind P, Brooks R, Rabin R, editors. *EQ-5D Concepts and Methods: A Developmental History*. 2005: pp 109–123. doi:10.1007/1-4020-3712-0_9

14. Pitkänen A, Välimäki M, Endicott J, et al. Assessing quality of life in patients with schizophrenia in an acute psychiatric setting: reliability, validity and feasibility of the EQ-5D and the Q-LES-Q. *Nord J Psychiatry*. 2012;66:19–25.

15. König H-H, Born A, Günther O, et al. Validity and responsiveness of the EQ-5D in assessing and valuing health status in patients with anxiety disorders. *Health Qual Life Outcomes*. 2010;8:47.

16. Van Der Zee CH, Priesterbach AR, Van Dussen L Der, et al. Reproducibility of three self-report participation measures: the ICF Measure of Participation and Activities Screener, the Participation Scale, and the Utrecht Scale for Evaluation of Rehabilitation–Participation. *J Rehabil Med*. 2010;42:752–757.

17. Post MWM, Van Der Zee CH, Hennink J, et al. Validity of the Utrecht Scale for Evaluation of Rehabilitation–Participation. *Disabil Rehabil*. 2012;34:478–485.

18. Chambless DL, Blake KD. Construct validity of the Perceived Criticism Measure. *Behav Ther*. 2009;40:155–163.

19. Hooley JM, Teasdale JD. Predictors of relapse in unipolar depressives: expressed emotion, marital distress, and perceived criticism. *J Abnorm Psychol*. 1989;98:229–235.

20. Cole JD, Kazarian SS. The Level of Expressed Emotion Scale: a new measure of expressed emotion. *J Clin Psychol*. 1988;44:392–397.

21. Gerlsma C, Hale WW. Predictive power and construct validity of the Level of Expressed Emotion (LEE) Scale. Depressed out-patients and couples from the general community. *Br J Psychiatry*. 1997;170:520–525.

22. Renshaw KD. The predictive, convergent, and discriminant validity of perceived criticism: a review. *Clin Psychol Rev*. 2008;28:521–534.

23. Hendrick SS. A generic measure of relationship satisfaction. *J Marriage Fam*. 1988;50:93–98. doi.org/10.2307/352430

24. Hendrick SS, Dicke A, Hendrick C. The Relationship Assessment Scale. *J Soc Pers Relat*. 1998;15:137-142. doi.org/10.1177/0265407598151009

25. Calvocoressi L, Mazure CM, Kasl S V, et al. Family accommodation of obsessive-compulsive symptoms: instrument development and assessment of family behavior. *J Nerv Ment Dis*. 1999;187:636–642.

26. Kreisman DE, Simmens SJ, Joy VD. Rejecting the patient: preliminary validation of a self-report scale. *Schizophr Bull*. 1979;5:220–222.

27. Manickam LSS, Chandran SR. Rejection of chronic schizophrenic patients : some preliminary observations from Kerala. *Indian J Psychiatry*. 1998;40:274–279.
